# Pneumothorax After VATS for Pleural Empyema in Pediatric Patients

**DOI:** 10.3390/children12020154

**Published:** 2025-01-28

**Authors:** Nariman Mokhaberi, Vasileios Vasileiadis, Jan-Malte Ambs, Konrad Reinshagen

**Affiliations:** 1 Department of Pediatric Surgery, University Medical Center Hamburg-Eppendorf, 20251 Hamburg, Germany; 2 Department of Pediatric Surgery, Hamburg Children’s Hospital Altona, 22763 Hamburg, Germany; 3 Paediatric Urology, Great Ormond Street Hospital for Children NHS Foundation Trust, London WC1N 3BH, UK; 4 Department of Pediatric Radiology, Hamburg Children’s Hospital Altona, 22763 Hamburg, Germany; 5 German Center for Child and Adolescent Health (DZKJ), Partner Site Hamburg, University Medical Center Hamburg-Eppendorf, 20251 Hamburg, Germany

**Keywords:** pneumothorax ex vacuo, VATS, pneumonia, necrotizing, children, pediatric, bronchopleural fistula

## Abstract

(1) Background: In children, bacterial pneumonia is the most common cause of parapneumonic pleural effusions which can eventually lead to pleural empyema. Treatment is varied and is a combination of antibiotic therapy, chest tube drainage, fibrinolytics and video-assisted thoracoscopic surgery (VATS). Postoperative complications of the latter include pneumothoraces and bronchopleural fistula (BPF). The aim of this study is to investigate the incidence and duration of pneumothoraces during the perioperative period and follow-up (FU) to elucidate their progression following video-assisted thoracoscopic surgery (VATS) to start to create an evidence-based standardized FU protocol. (2) Methods: This retrospective study included all patients who underwent VATS for pleural empyema between January 2013–May 2023 at the University Medical Center Hamburg-Eppendorf (UKE) and the Hamburg Children’s Hospital Altona (AKK). (3) Results: We identified 47 patients with pleural empyema who underwent VATS. A proportion of 43% of patients were found to have a pneumothorax with 55% of those being unresolved at discharge. At the end of FU, 27% of those had a “pneumothorax ex vacuo”. No surgical interventions were needed. (4) Conclusions: The majority of pneumothoraces after VATS in pediatric patients can be managed conservatively. In the context of follow-up care, it is recommended that X-ray examinations should be used sparingly, while sonographic follow-up examinations should be conducted more frequently. If the pneumothorax persists, further thoracoscopy for resection of the visceral pleura and treatment of bronchopleural fistula may be the next step in treatment.

## 1. Introduction

In children, bacterial pneumonia is the most common cause of parapneumonic pleural effusions, which can eventually lead to pleural empyema. Empyema is also closely linked to necrotizing pneumonia (NP) which is most commonly caused by *Pneumococci* and *Staphylococcus aureus*, often accompanied by bronchopleural fistula (BPF) [1]. In addition to pneumonia, other causes include blunt or penetrating chest trauma, infected congenital cysts or mediastinitis. Pleural empyema is characterized by purulent secretions with pleural inflammation within the pleural space and can be described in the following stages: exudative, fibrinopurulent and organizing [2,3].

As pediatric patients rarely have a primary lung disease, the prognosis in children is excellent and the majority of patients make a complete recovery. However, pleural empyema remains an important cause of morbidity [2,4].

Treatment usually starts with intravenous antibiotic therapy, analgesia, antipyretics and, if necessary, oxygen supplementation. If possible, antibiotic treatment should be adjusted according to microbiological results. In cases of pleural effusion that impairs lung function, a pigtail or large drain can be inserted. Furthermore, the British Thoracic Society (BTS) guidelines for the management of pleural infections in children recommend the use of fibrinolytics. Fibrinolytics have been shown to reduce the length of hospital stay and to aid filtration and reabsorption of pleural fluid [4].

The role and timing of surgery are still controversial and depend on the individual clinic and the age of the patient. However, there is agreement that a multidisciplinary approach to treatment benefits patient outcomes. Surgery should be considered when initial treatment with antibiotics, chest tube drainage and possibly fibrinolytics has failed [3].

Early VATS (video-assisted thoracoscopic surgery) improves the chances of full lung expansion and adequate drainage of the pleural effusion in pediatric patients. However, its efficacy is supported by limited evidence due to the absence of large prospective studies [5,6,7,8].

Potential severe complications of empyema include bronchopleural fistula and the formation of a pneumothorax or eventually a “pneumothorax ex vacuo” [9,10]. A “pneumothorax ex vacuo” is a consequence of fibrinous tissue formation between the visceral and pleural tissue that traps the lung or individual lobes, which restricts lung expansion. This is also a recognized complication of NP, where the pleural peel induces a high negative pressure within the affected chest [1]. The elevated pressure may lead to air leakage into the pleural cavity, resulting in a “pneumothorax ex vacuo”. This phenomenon often occurs during the chronic inflammatory phase of pleural tissue healing. Most patients are asymptomatic and the condition is typically managed conservatively through observation [11,12,13].

For pleural empyema, the BTS recommends clinical and radiological follow-up for at least 4–6 weeks after discharge. Beyond this, further investigations will depend on the patient’s general health and radiological appearance. For BPF, no clear consensus currently exists for treatment in adult or pediatric patients. Treatment options range from conservative management, with ventilation adjustments, prolonged chest tube drainage and targeted use of water seal, to chemical or autologous blood patch pleurodesis, endobronchial valves or stent implantation and surgical re-intervention with stump revision or muscle flaps [14,15,16,17,18].

It has been shown that the majority of chest radiographs do not demonstrate residual disease after 3–6 months. However, the existing literature does not explicitly address the clinical development of pneumothoraces in its results [11,12,13,19,20,21].

The pivotal feature of this study is therefore to examine the incidence of pneumothoraces in pediatric patients who have undergone VATS for pleural empyema both in the perioperative period and during follow-up. The goal of this study is to validate the existing scientific knowledge in order to adjust the follow-up plan accordingly and avoid unnecessary interventions for children wherever possible.

## 2. Materials and Methods

### 2.1. Patient Identification

**Figure 1:**
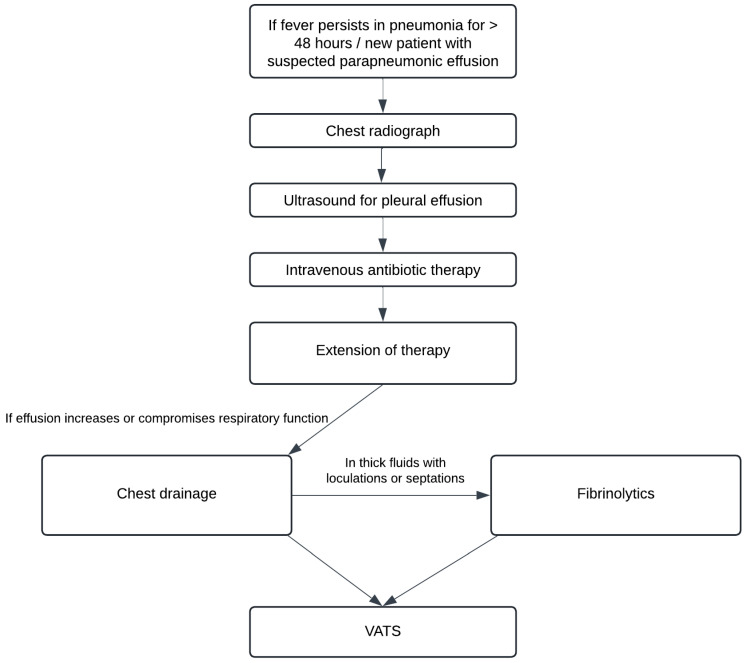
Algorithm for the management of pleural infection in children.

This retrospective study included all patients who underwent VATS for pleural empyema between January 2013 and May 2023 at the Departments of Pediatric Surgery at the University Medical Center Hamburg-Eppendorf (UKE) and the Hamburg Children’s Hospital Altona (AKK). Pleural empyema was diagnosed sonographically or via CT/MRI in accordance with standard protocol when a pleural effusion was identified on chest X-ray (Figure 1). A microbiological examination of the punctate was conducted either when a chest drain was inserted or as part of VATS.

### 2.2. Ethical Consideration

The study adhered to the principles outlined in the Helsinki Declaration and received approval from the local institutional review board, the Hamburg Ethics Committee (2023-101176-BO-ff).

### 2.3. Study Protocol

The medical patient records were retrospectively analyzed looking at demographic and surgical data as well as information on length of stay (LOS), time from surgery to discharge and time from admission to surgery was acquired. The primary endpoint was the occurrence and resorption of pneumothoraces. Secondary endpoints included LOS, insertion of a drain, performance of a CT or MRI scan, microbiological findings, use of fibrinolytics, duration of follow-up (FU; defined as time from discharge to last outpatient appointment), number of chest radiographs during FU, residual “pneumothorax ex vacuo”.

### 2.4. Statistical Analysis

Statistical analysis was carried out using Microsoft Excel for Mac (Version 16.59) and Prism 9 for macOS (Version 9.5.0). The significance level was set at *p* < 0.05 for all statistical analyses. Descriptive statistics are presented in both total numbers and relative percentages. Continuous variables were checked for deviation from normal distribution (Shapiro–Wilk test) for each comparison. Continuous variables are presented as median with interquartile range and categorical variables were expressed as frequencies (n) and percentages (%). Percentages were rounded to whole numbers. Differences between groups were calculated using the Mann–Whitney U test for nonnormally distributed data.

## 3. Results

A total of 166 patients with pleural empyema were treated at the UKE and AKK from January 2013 to May 2023. Of these, 47 patients underwent VATS (28%). The patients comprised 24 males and 23 females. The median age was 4 years (IQR = 5), with the youngest patient aged 2 and the oldest aged 17 (Table 1).

The data relating to the inpatient course can be found in Table 2. The mean follow-up period after hospital discharge was 60.49 days. During FU, a median of one chest radiograph was taken (IQR = 1.3). X-rays were not taken after discharge in 14 patients.

**Figure 2:**
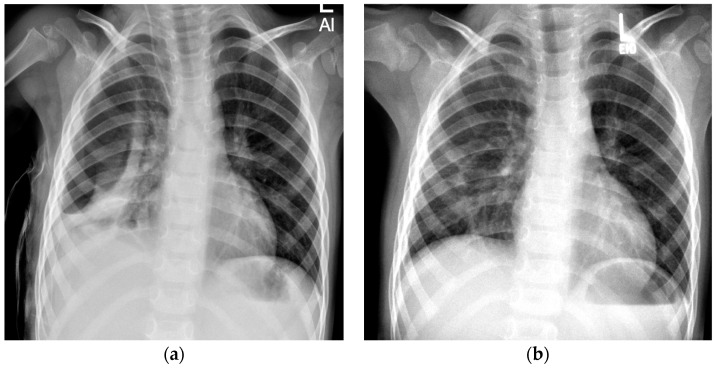
Spontaneous resorption of a pneumothorax in a 3-year-old boy. (**a**) Anterior–posterior (AP) chest radiograph taken at the time of discharge reveals a right-sided pneumothorax. (**b**) Spontaneous resorption of the pneumothorax at 2-month follow-up.

**Figure 3:**
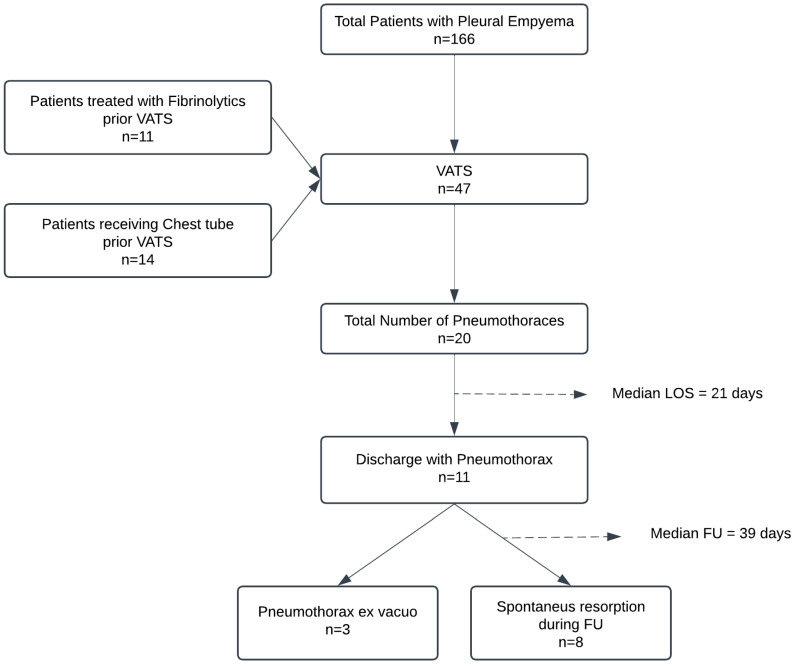
Treatment process of the study population.

During our inpatient treatment, a pneumothorax was detected in 20 out of 47 patients (43%). Pneumothorax was still detectable in 11 patients at the time of their discharge from inpatient treatment. At the end of our FU, a spontaneous resorption of the pneumothorax was observed in eight patients (Figure 2). In three patients, a pneumothorax ex vacuo remained identifiable. The course of treatment is shown below (Figure 3).

In 30 patients, an CT scan was conducted prior to the surgery, while two patients underwent an MRI scan before surgery. The indications for performing a CT or MRI scan included the absence of clinical improvement during ongoing treatment, as well as further diagnostic imaging in the case of pleural effusions.

A total of nine cases of BPF were documented during the inpatient course. Of the patients, only one received a chest tube prior to VATS. This patient also developed BPF before VATS. The remaining patients developed BPF after VATS. One patient underwent Re-VATS 15 days after the initial surgery due to persistent empyema and BPF. Since the fistula persisted and the affected lung was not adequately expanded, a thoracotomy was performed 8 days later in which the BPF was sutured using PDS 4/0 (Ethicon Inc., Raritan, NJ, USA). All other cases of BPF were treated conservatively.

Microbial evidence of bacteria was detected in the pleural fluid, by polymerase chain reaction or in histological findings in 21 patients (45%), with *Streptococcus pneumoniae* being the most frequently identified bacteria in 12 cases. In 26 patients, no bacteria could be identified. Fibrinolytics were administered to 11 patients (23%). The administration of fibrinolytics was performed when there was a progressive clinical deterioration despite the prior administration of antimicrobial therapy or chest drainage. The prescribed fibrinolytics included Urokinase, Dornase alfa (Pulmozyme), Alteplase and DNase.

There was no significant difference in the duration of hospital stay between patients treated with fibrinolytics first or who underwent drainage prior to VATS and those treated directly by VATS (Table 3).

## 4. Discussion

Parapneumonic pleural effusions and pleural empyema are among the major complications of pediatric pneumonia. There is a consensus that therapy for pleural empyema involves antibiotic treatment, and may also include administration of fibrinolytics, drainage or VATS [18]. Possible complications include the formation of pneumothoraces or the development of BPF.

VATS for pleural empyema is a challenging procedure with a significant risk of complications; but with overall low morbidity and mortality [22]. Our results indicated that the aforementioned complications occurred cumulatively in almost half of the patients but also demonstrated that the majority of pneumothoraces following VATS in pediatric patients resolved spontaneously without intervention. These findings align with the basic understanding of spontaneous resorption of pneumothoraces or a “pneumothorax ex vacuo”. Both Chan et al. and Gocmen et al. showed that the majority of chest radiographs were normal after three to six months [11,12,13,19,20,23]. Also, Virkki et al. showed that routine chest X-rays are not necessary for conservatively treated community-acquired pneumonia in asymptomatic children [24]. In addition, Gursoy et al. determined that in the context of low-grade empyema, the performance of routine X-ray examinations is not indicated in asymptomatic patients during the follow-up period. This conclusion was based on the observation that, in their study, 80% of pleural thickening after pleural empyema was no longer detectable within five months [25]. In a prospective study of 30 children with pleural empyema, 60% of whom underwent surgery, it was demonstrated that radiological residuals were still visible in only one patient at the four-month follow-up. In contrast to the characteristics of our group, surgical treatment was performed via open thoracotomy [26]. Furthermore, the results of a prospective study demonstrated that lung ultrasonography has comparable diagnostic efficacy and reliability in detecting pleural effusions in children with clinically suspected pneumonia to that of conventional X-ray [27]. These findings support our conclusion and those of previous studies on reducing the amount of chest radiographs during follow-up of pleural effusions and empyema and to perform thorax ultrasound primarily [28]. The recommended routine clinical and sonographic follow-up can also be more effectively implemented in low-income countries through the progressive use of “Clinician-performed-point-of-care ultrasound” (POCUS) in resource-limited settings with limited access to radiological imaging [29].

Furthermore, only one patient with BPF required surgical intervention; therefore, conservative management has been shown to be effective in almost all of our cases of BPF (8/9, 88%), avoiding the need for surgical intervention. This finding contrasts with the more invasive approaches often recommended in adult patients or in children as previously described by Jester et al. or the BTS [4,14]. Other described approaches include pleurodesis and interventional endobronchial valves or tubes [16]. In support of our results, McKee et al. reported 16 cases of bronchopleural fistula out of a total of 307 cases with BPF in a retrospective 8-year review. Of those patients, only five required surgical intervention by VATS, while the rest of the air leaks resolved [30].

The literature on this mainly consists of case studies with the majority of studies describing spontaneous resorption in patients with an ex vacuo pneumothorax, indicating that no intervention is necessary [31,32,33].

At the end of our FU, three patients showed to have a “pneumothorax ex vacuo”. It should be noted that one of the two patients with a “pneumothorax ex vacuo” at the end of our FU period was immunosuppressed due to liver transplantation for progressive familial intrahepatic cholestasis. After discharge from our inpatient treatment, there was no renewed X-ray control, although the patient was regularly treated due to his underlying disease. The second patient was also not subjected to an X-ray during the follow-up period. Instead, a clinical and sonographic examination was performed.

**Figure 4:**
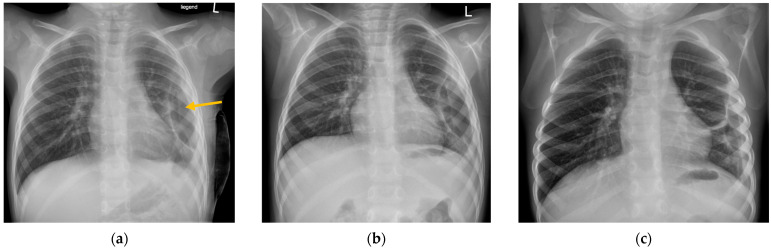
“Pneumothorax ex vacuo” in a 2-year-old girl. (**a**) Anterior–posterior (AP) chest radiograph taken 2 days after discharge showing a left-sided pneumothorax (arrow). (**b**) Persistent pneumothorax 7 days after discharge. (**c**) At 2.5 weeks, pneumothorax is still radiologically detectable.

The third patient with a “pneumothorax ex vacuo” was transferred to the UKE from an external hospital. At the time of transfer, the patient had been symptomatic for 11 days and was receiving intravenous antibiotic therapy. After transfer, a pleural drain was initially inserted, which had to be revised the following day due to dislocation. In the course of the procedure, the drain was pulled accidentally, resulting in a progressive pneumothorax, so that VATS was performed and the drain was reinserted. Therefore, this patient had three thoracic interventions. After the patient was discharged, there were a total of three follow-up radiological examinations within 3 weeks, which still showed a detectable but regressive “pneumothorax ex vacuo”. After the last follow-up, the patient returned to her home country and was not available for further follow-up (Figure 4). It is important to highlight that the FU period for these two patients was shorter than our average follow-up period, and with a longer follow-up period detection of spontaneous reabsorption of the pneumothorax might have been seen in these cases as well.

The case of another patient, who is not included in our cohort because he had VATS as a secondary intervention, should nonetheless be highlighted due to its relevance.

The 3-year-old boy was transferred to our hospital with a septated pleural empyema. A pigtail drain was placed; initial antibiotic treatment with ampicillin/sulbactam was extended with gentamicin after microbiological detection of MRSA. An apical pneumothorax was noted as complication of the drain, which remained stable with subsequent treatment as an inpatient. The patient was discharged 12 days after admission to the hospital, but follow-up examinations over the next 4 months showed that the pneumothorax had progressed, resulting in a shift of the mediastinum. The patient was symptomatic, therefore thoracoscopy was indicated; VATS with pleural lysis and partial pleurectomy was performed. Subsequently, regular lung expansion was observed without further complications and the patient was discharged after one week.

With regard to the secondary endpoints, the average time from surgery to discharge for patients treated solely with VATS in our study was longer than in recent studies [34,35]. However, this may also be due to different treatment procedures in the respective countries and does not necessarily result solely from the clinical course.

Fibrinolytics were only administered to 23% of patients prior to undergoing VATS. However, as has previously been demonstrated, treatment involving drainage and fibrinolytics is not inferior to treatment with VATS with regard to hospitalization or the time until the patient is free of fever. Drainage plus fibrinolytics instillation is therefore considered to be the first-line treatment. This finding is corroborated by a recent review by Fernandez Elviro et al., which also favors the primary use of a chest tube and fibrinolytics, demonstrating comparable clinical outcomes and a reduced length of stay (LOS) when compared with thoracotomy or VATS while incurring a lower financial burden [22,36,37].

Additionally, almost one-third of the patients received chest tube drainage before VATS. There were no significant differences in LOS between patients who received chest tube drainage or fibrinolytics before VATS.

A review including randomized controlled trials on the management of pleural empyema in all age groups showed that VATS may reduce LOS compared to chest tube drainage alone [38]. Our finding is possibly due to our selection criteria, since all patients in our study received VATS treatment. These cases represent the most severe forms of pleural empyemas, requiring surgical intervention despite prior use of fibrinolytics or pleural drainage. In addition to an ultrasound or X-ray examination, a CT or MRI scan was performed in 32 patients. This is higher than the figures reported in the literature. It is important to note that the results may be influenced by the varying availability and treatment strategies employed in the countries where the studies were conducted, as well as the severity of the disease in our study [6,10,23,35,39].

The study may be limited due to missing data or imprecise accuracy in the charts resulting from the retrospective nature of the study; therefore, this restricts the conclusions that can be drawn. The interpretation of small residual pneumothoraces and the objectification and reliable detection of BPF in non-electrical chest drainage systems also harbors potential sources of error. Additionally, five cases of pneumothorax were identified prior to the surgical procedure. In these cases, it can be discussed whether a conservative spontaneous resorption actually occurred in the FU or whether the resorption was more likely to have been surgical in the context of video-assisted thoracoscopic surgery (VATS). However, all five cases also showed a pneumothorax postoperatively, meaning that it is ultimately not possible to clearly differentiate between the two and thus speak of a mixed picture.

Moreover, the treatment algorithm was not strictly followed in all patients but was adjusted during the follow-up period for those who no longer required radiology. Furthermore, it has been demonstrated that the actual follow-up period was shorter than required by internal standards. It should also be added that cross-sectional imaging would be required to rule out residual findings such as “pneumothorax ex vacuo” or pneumatocele definitively. However, this remains a theoretical approach for discussion, especially in pediatric patients.

## 5. Follow-Up Algorithm

**Figure 5:**
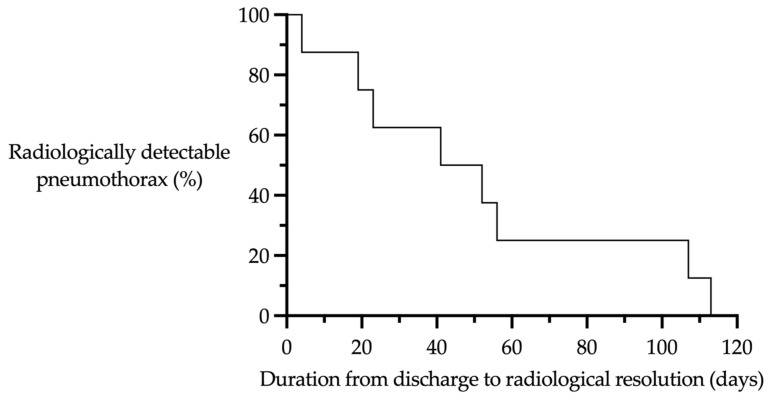
Radiological detectability of the pneumothorax during the FU.

**Figure 6:**
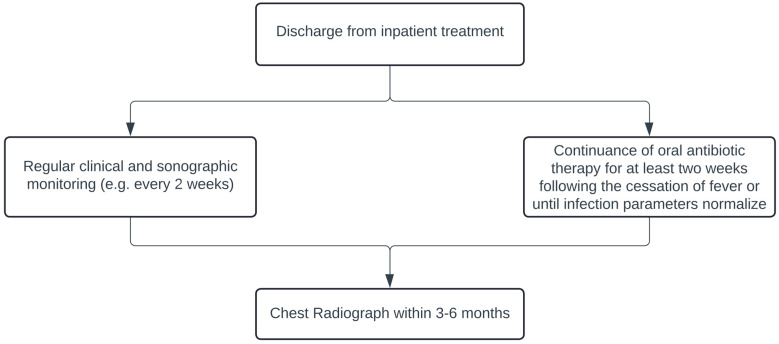
Follow-up algorithm after patient’s discharge from hospital.

Drawing upon our experience, we advocate regular clinical and primarily sonographic monitoring, at the latest every two weeks, though always adapted to the clinical situation. Oral antibiotic therapy is continued for a period of at least 2 weeks following the cessation of fever or until infection parameters normalize [4,40]. A chest X-ray is recommended within 3–6 months, although the data demonstrate that the pneumothorax was absorbed earlier on average in this cohort as the average time from discharge to “radiological resorption” of the pneumothorax was 52 days (median: 47 days) (Figure 5). Further X-ray examinations should be carried out depending on the findings. Additive measures in the outpatient setting, such as additional oxygen administration or physiotherapy, are not standard; however, there are data indicating that these measures can support the healing process [41,42] (Figure 6).

## 6. Conclusions

To the best of our knowledge, this study is the first to comprehensively evaluate the clinical course and follow-up of pneumothoraces following VATS in pediatric patients with pleural empyema. The primary objective was to enhance understanding of pneumothorax progression and optimize postoperative management strategies.

Our findings indicate that pneumothoraces after VATS in pediatric patients for pleural empyema demonstrate a high rate of spontaneous resorption, with most patients remaining asymptomatic. Based on these results, conservative management of residual pneumothorax is strongly recommended and routine chest radiographs are deemed unnecessary in asymptomatic cases. This finding is consistent with the results of previous studies that have investigated the clinical and radiological convalescence of children with pleural empyema following both conservative and surgical treatment. Similarly, the presence of BPF with pleural empyema should be managed conservatively in asymptomatic pediatric patients.

We support that follow-up care should emphasize regular clinical evaluations and ultrasonographic monitoring as standard practice. However, ultrasonography is more appropriate for the monitoring of effusions than pneumothoraces, thus underscoring the continued significance of the targeted utilization of X-rays. The use of multiple chest radiographs should be minimized, except in special cases warranting further investigation. In instances where pneumothoraces persist, increase in size or are associated with clinical symptoms, additional thoracoscopy may be required to resect fibrotic visceral pleura and address any underlying bronchopleural fistula. Conducting prospective studies with a standardized protocol on sonographic FU and chest radiographs is imperative to validate the findings and recommendations.

## Figures and Tables

**Table 1:** Demographic characteristics and clinical parameters.

	*n* (%)
Gender	
Male	24 (51)
Female	23 (49)
Pneumothorax	
Yes	20 (43)
Preoperative diagnosis	5 (11)
Postoperative diagnosis	15 (32)
No	27 (57)
Pneumothorax at discharge	
Yes	11 (55)
No	9 (45)
Pneumothorax ex vacuo	
Yes	3 (6)
No	43 (92)
Unclear	1 (2)
Bronchopleural fistula	
Yes	9 (19)
No	38 (81)
Chest tube drainage prior to VATS	
Yes	14 (30)
No	33 (70)
Fibrinolytics prior to VATS	
Yes	11 (23)
No	36 (77)
CT/MRI Scan	
Yes	32 (68)
No	15 (32)
Microbial findings	
Strep. pneumoniae	12
Strep. hominis	1
Kleb. pneumoniae	1
Strep. pyogenes	2
Strep. mitis	1
Strep. intermedius	1
Staph. aureus	3
Influenza virus	1
Human metapneumovirus	1

**Table 2:** Inpatient course and follow-up period.

	Median (IQR)
Length of patients’ hospitalization (days)	21 (16)
Median duration from hospitalization to surgery (days)	4 (8)
Median duration from surgery to discharge (days)	15 (9)
Median FU period (days)	39 (109)
Median number of chest radiographs during FU	1 (1.3)

**Table 3:** Length of stay (LOS).

	**Chest Tube Prior to VATS (*n* = 14)**	**No Chest Tube Prior to VATS (*n* = 33)**	***p***
LOS (median, days)	27	20	0.0547
	**Fibrinolytics Prior to VATS (*n* = 11)**	**No Fibrinolytics Prior to VATS (*n* = 36)**	***p***
LOS (median, days)	23	21	0.3346

## Data Availability

The data described in this study are accessible from the corresponding author upon request. Due to ethical and private constraints, the data are not publicly available.
